# Ethical allocation of physical and occupational therapy in acute care

**DOI:** 10.1002/jhm.70111

**Published:** 2025-06-23

**Authors:** Maylyn Martinez, William F. Parker

**Affiliations:** ^1^ Section of Hospital Medicine University of Chicago Chicago Illinois USA; ^2^ Division of the Biological Sciences University of Chicago Chicago Illinois USA

## INTRODUCTION

Hospitalization is an important precipitant of disability. Adults who are hospitalized are 60% more likely to develop long‐term disability than those who are not^1^ and hospital‐acquired disability (HAD) occurs in approximately one‐third of all hospitalized patients.^2^ Many patients who experience HAD require discharge to a skilled nursing facility for rehabilitation, 13% being institutionalized for the first time in their lives.^3^ Many patients who develop HAD will have permanent disability^4^ requiring long‐term assistance with activities of daily living (ADL)^4^ and increased use of community resources.^5^, ^6^ Studies have repeatedly shown that physical therapy (PT) is a key component of the treatment and prevention of HAD and mobility loss.^7^, ^8^, ^9^ Compared to patients who have fewer than three PT sessions during hospitalization, those who receive three to four sessions have 20% more functional improvement and are 4% more likely to discharge to home instead of a nursing facility. For those receiving more than seven sessions, function improves by 78% and likelihood of discharge home by 22%.^10^ There is a growing large body of evidence that supports these findings. While patients admitted to the hospital with irreversible pre‐existing functional impairments or total functional independence can benefit from simple nursing interventions or independent ambulation, we know that those with new functional impairments or at high risk for HAD will have better functional and discharge outcomes with more in‐hospital physical rehabilitation.

The American Physical Therapy Association reported that in 2022, there were 5.2% fewer therapists than needed to meet demand, and that number would grow to 14.7% by 2037. Unfortunately, despite the enormous benefits of physical and occupational therapy (OT) for certain patients, these staffing shortages and the absence of mandated staffing ratios for therapists severely limits their supply in hospitals. Because of clear associations with care quality and patient safety, the Center for Medicare and Medicaid Services (CMS) mandates that hospitals have adequate numbers of licensed registered and vocational nurses but, even for them, does not mandate specific staffing ratios. Instead, factors including patient acuity, admission numbers, and staff expertise and skills are used to make nurse staffing decisions on a unit‐to‐unit basis.^11^, ^12^ For physical and occupational therapists working in the acute care setting, there is no legislation for staffing, let alone mandates for specific staffing ratios. Little is known about the basis for therapy staffing decisions or what role hospital leadership expects them to fill, but studies show that, based on the referrals they receive, therapists feel they are frequently mistaken as discharge planners or “a walking service,”^13^ rather than experts in physical rehabilitation. This was underscored in a study showing that as many as 38% of PT referrals in hospitalized patients are for those without mobility limitations who are at low risk of hospital‐acquired disability.^14^ Based on these findings, it is likely that referring providers consider uninformative factors (or fail to make their own assessment) before deciding whether to refer a hospitalized patient for PT/OT.

**Figure 1:**
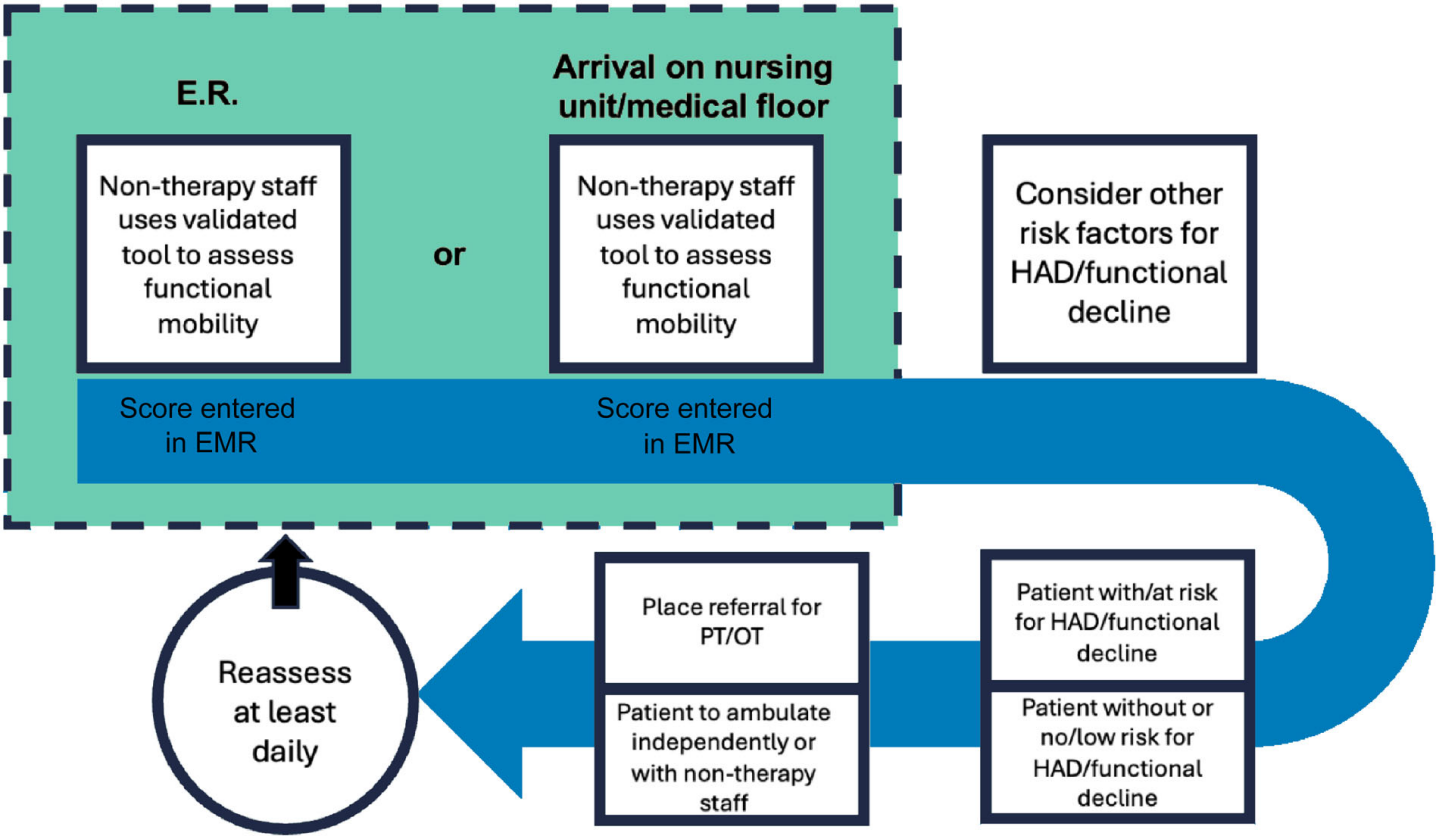
Conceptual framework for PT and OT allocation in patients hospitalized for medical illness. Nontherapy staff refers to nurses or nursing assistants, mobility aides or assistants, and so on. Other risk factors for HAD/functional decline include older age, Black race, and social disadvantage. E.R., emergency room; EMR, electronic medical record; HAD, hospital‐acquired disability; OT, occupational therapy; PT, physical therapy.

While physical function should be the primary criterion driving rehabilitation referrals, there are other valid reasons to refer given therapists' broad scope of practice (e.g., a specific need to evaluate stairs for an otherwise high‐functioning patient, specialized assessments for vestibular function, or addressing unique barriers to discharge). However, current PT/OT referral practices lack specificity. This results in therapists performing large numbers of unnecessary evaluations, which shifts this invaluable resource away from patients who truly need rehabilitation to give them their best chance at a home discharge and prevention of permanent disability. A better strategy would be to start incorporating existing evidence and tools into our therapy referral decisions to help ensure fair and appropriate allocation of resources (Figure 1).

## VALIDATED TOOLS TO IDENTIFY WHO WILL BENEFIT FROM INPATIENT PT AND OT

In recent years, rehabilitation experts have developed and validated objective tools for evaluating functional mobility and ability in hospitalized patients. There is growing utilization of the Johns Hopkins Highest Level of Mobility (JH‐HLM) scale and the Activity Measure Post‐Acute Care (AM‐PAC) Inpatient Mobility and Activity Short Forms. These tools are currently in use in hospitals across the country. They relay essential information to the healthcare team about a patient's mobility and functional independence: JH‐HLM distinguishes between patients who can achieve a range of performance goals from lying in bed to walking 250 feet or more; The AM‐PAC basic mobility short form assesses difficulty and help needed with six basic activities: turning over in bed, standing up from a chair, transitioning from lying to sitting, transferring between bed and chair, walking in the hospital room, and climbing 3–5 steps. A separate AM‐PAC assesses difficulty and help needed with six ADLs (dressing, bathing, toileting, etc.) Implementation and familiarity with these tools allow a patient's medical team to discuss and make decisions about their mobility needs for the day as well as discharge planning. At hospitals like Johns Hopkins Medicine and UChicago Medicine, mobility assessments for hospitalized patients are performed by non‐therapist clinicians (e.g., nurses) using these tools. Those assessments are then used to help guide therapy referral decisions and track patients' mobility progress during admission. Studies have shown that this strategy reduces consults for patients at the lowest risk for functional decline.

## EVIDENCE‐BASED PREVENTION OF HOSPITAL‐ACQUIRED DISABILITY MAY MITIGATE HEALTH INEQUITIES

While baseline functional impairments and older age are known risk factors for HAD, there is evidence that Black race and social disadvantage also confer increased risk. Black patients with dementia admitted to hospitals have lower physical function^15^ and among community‐dwelling Medicare beneficiaries, Black and Hispanic patients were more likely to develop mobility limitations and ADL difficulties over time.^16^ Among patients with peripheral artery disease, Black patients are 44% more likely to experience mobility loss and 67% more likely to experience functional decline than White patients.^17^ Social disadvantage at the neighborhood level has also been associated with a 9% higher disability burden over the 12 months following ICU discharge.^18^ Despite these functional disadvantages, Black patients and those with social disadvantage are shown to have lower rates of utilization of PT.^19^ The exact mechanisms for these disparities remain unknown but likely include multiple levels of influence such as individual (e.g., treatment preferences), interpersonal (e.g., explicit discrimination, implicit bias), or societal (e.g., quality of care). Given this, hospitalists can help close some of these gaps by considering race and social disadvantage risk factors for HAD and functional decline during hospitalization when making the decision to refer to PT or OT. Validated tools to understand precisely how to weigh social determinants of health into the PT/OT referral decision are still in development. In the meantime, awareness of these health inequities may serve as a guide.

## PATIENTS, PAYORS, HOSPITALS, AND CLINICIANS AGREE: THE GOAL IS DISCHARGE TO HOME

Nearly $60 billion in Medicare spending for postacute care services occurred in 2022.^20^ Thus, there has been discussion of how to decrease healthcare expenditures in this area. Moreover, discharge‐to‐home is a highly sought‐after goal for hospitals given it decreases length of stay, makes beds available for incoming patients, and is also an important component of rankings such as the US News and World Report Best Hospitals. Patients also have a strong preference for returning home after hospitalization. Current hospital payment models bundle inpatient therapy services into diagnosis‐related groups (DRGs). This means therapists do not bill separately for professional services, a structure that may disincentivize hospitals from hiring more physical therapists. However, the current body of evidence points to an association of increased PT/OT during hospitalization with improved functional outcomes and increased likelihood of discharge to home. Therefore, patient, payor, and hospital goals align in this regard. Furthermore, PT and OT play a key role in identifying those patients that will not achieve the functional recovery required to discharge to home, which enables timely discharge planning and efficient discharge to postacute care facilities. Hospitals should prioritize hiring more physical and occupational therapists and allocate therapy strategically to those who need it taking into consideration known risk factors like age, baseline function, race, and social disadvantage. This represents a strategy that can achieve both economic and health equity goals.

In summary, evidence‐based allocation of PT and OT can prevent hospital‐acquired disability and functional decline. Getting these services to the right people at the right time during hospitalization is not only operationally efficient but also ethical and just. Allocation can be improved by understanding that age, physical function, race, and social disadvantage are all risk factors for functional decline and HAD. Communicating to hospital leadership the advantages of intentional allocation of PT and OT is of utmost importance if we are to decrease functional decline and disability equitably and for all patients.

## CONFLICT OF INTEREST STATEMENT

The authors declare no conflict of interest.
